# Supplementary figures and images for: Dynamical model of the CLC-2 ion channel reveals conformational changes associated with selectivity-filter gating
Source: PLoS Comput Biol. 2020 Mar 30;16(3):e1007530. doi: 10.1371/journal.pcbi.1007530 (PMC7145265; doi:10.1371/journal.pcbi.1007530)

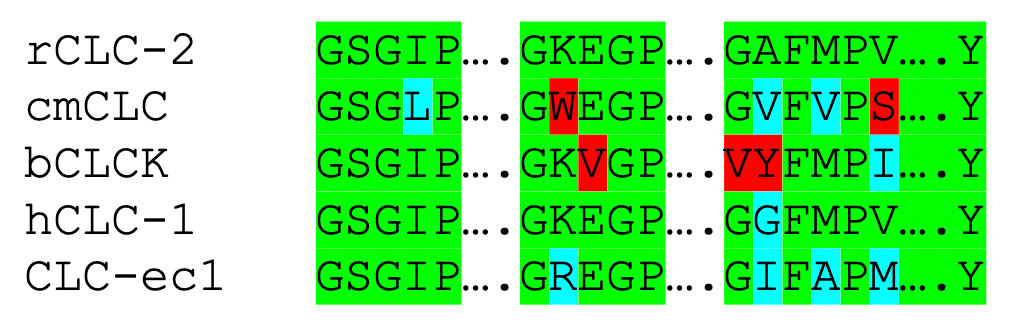

Supplement: S1 Fig — (TIF) [file pcbi.1007530.s001.tif]

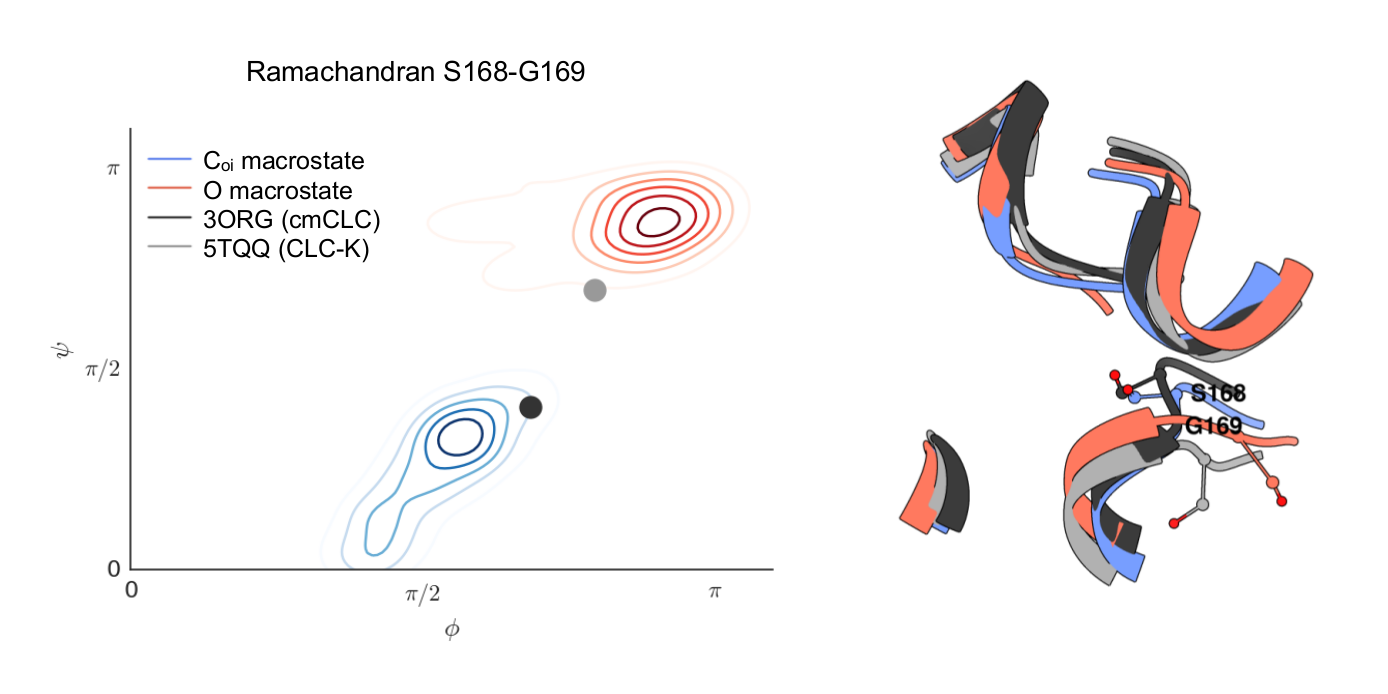

Supplement: S2 Fig — Sample SF conformation (right) for each structure or set of structures depicted in the Ramachandran plot. From our macrostates we are able to sample many conformations. These conformations represent the conformational diversity obtained from simulation techniques. This is evidenced by the wells in the Ramachandran plot. The contour lines represent the probability density, where darker the lines denote higher probability conformations. We see that the O and Coi macrostates represent distinct conformational wells. The template cmCLC structure, 3ORG, rests at the edge of the Coi macrostate, while the CLC-K structure, 5TQQ, rests at the edge of the O macrostate. (TIF) [file pcbi.1007530.s002.tif]

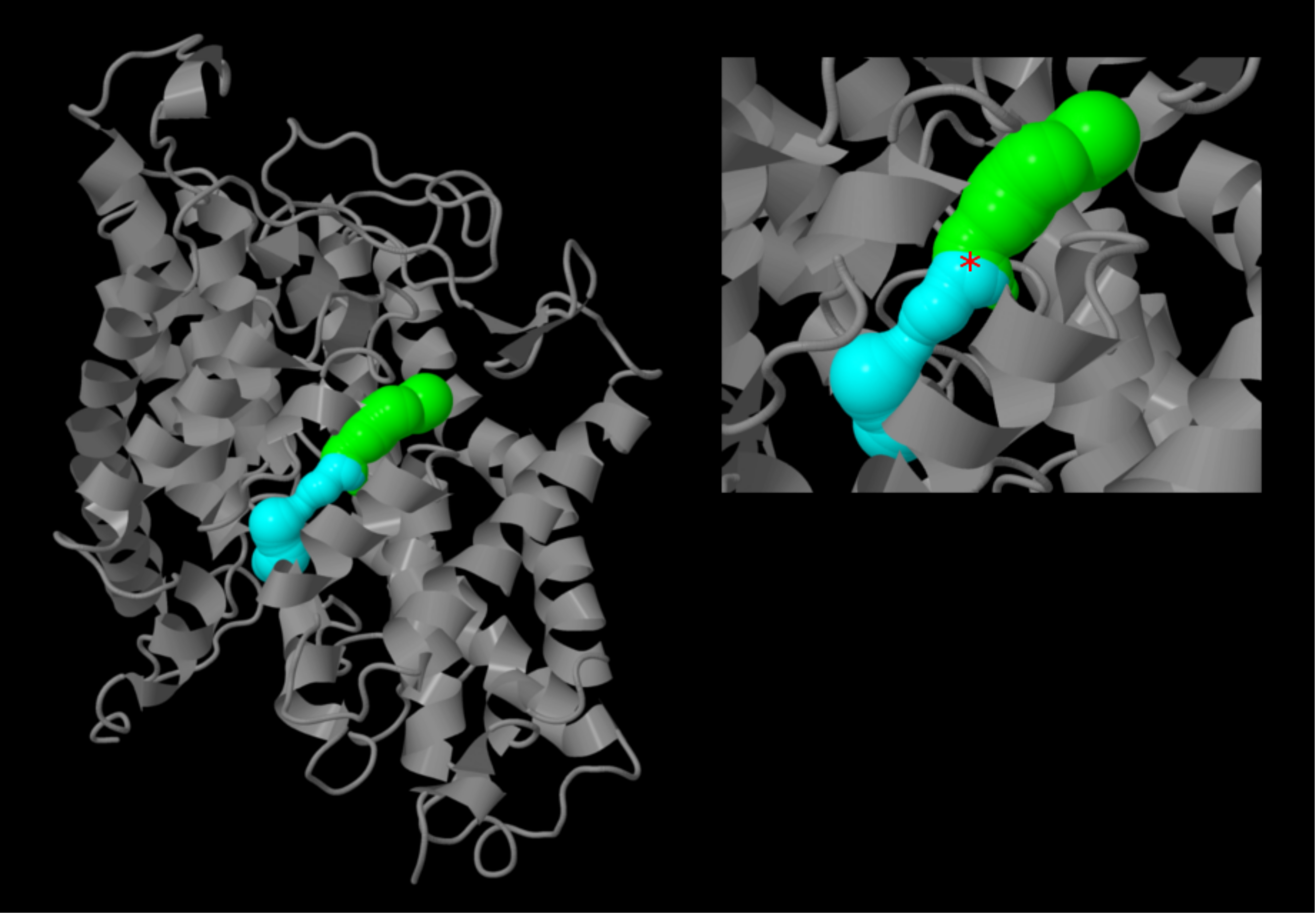

Supplement: S3 Fig — Full structure is depicted on left, bottleneck region at Sext (denoted by the red asterisk) is depicted in the inset. (TIF) [file pcbi.1007530.s003.tif]

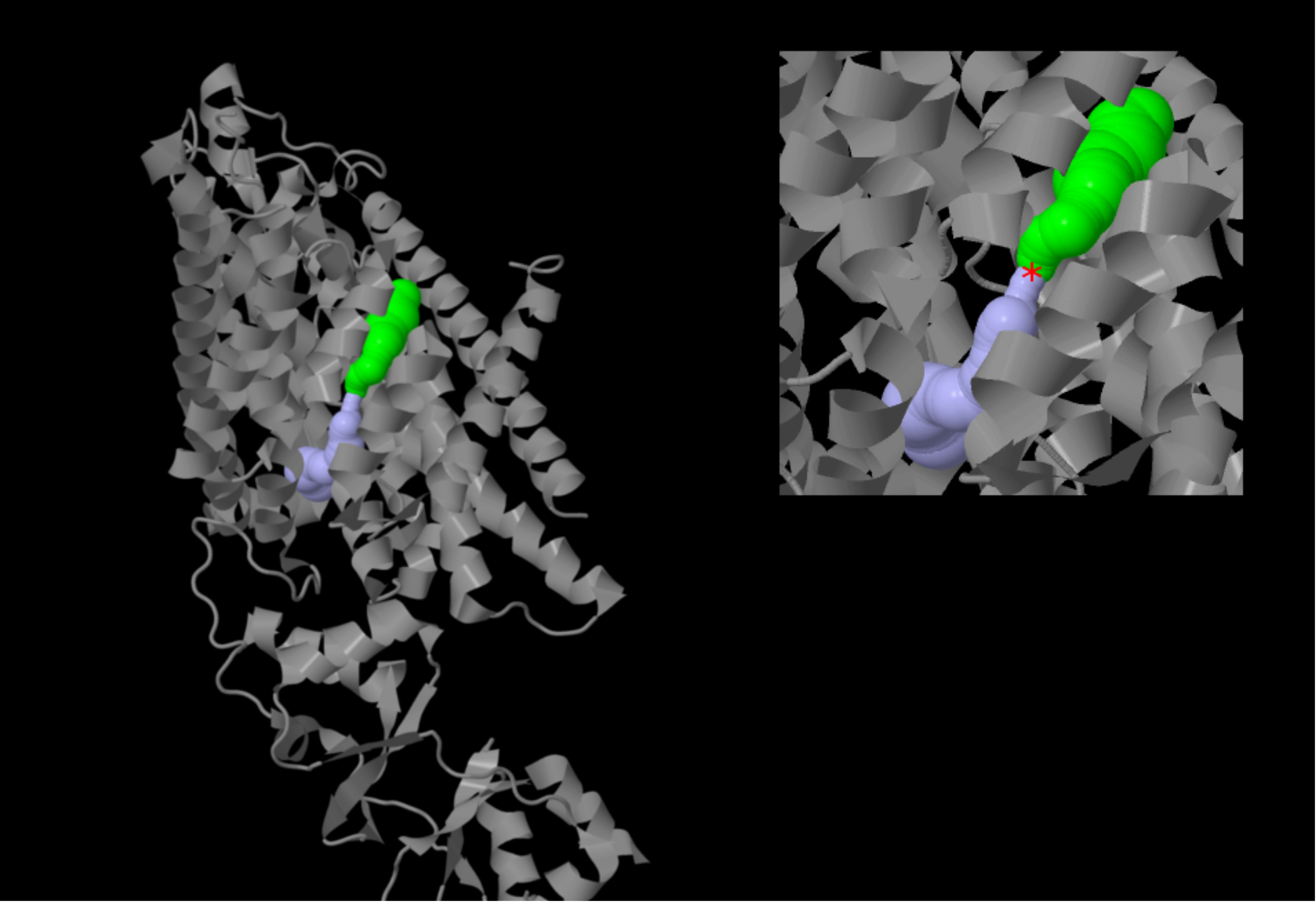

Supplement: S4 Fig — Full structure is depicted on left, bottleneck region at Sext (denoted by the red asterisk) is depicted in the inset. (TIF) [file pcbi.1007530.s004.tif]

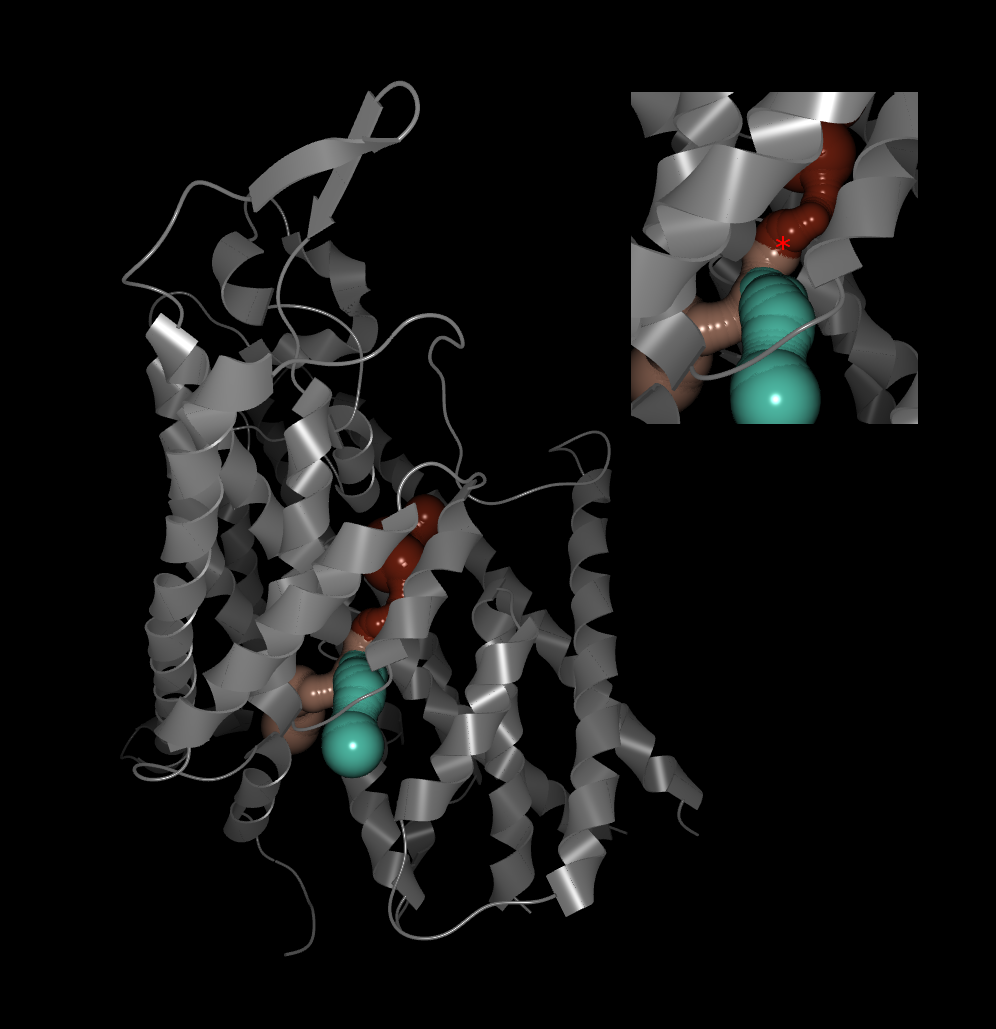

Supplement: S5 Fig — Full structure is depicted on left, bottleneck region at Sext (denoted by the red asterisk) is depicted in the inset. Note that in the main body of the manuscript, the radii of the dark red extracellular and the brown intracellular pathways are examined. This intracellular pathway was chosen for analysis because it was the wider of the two paths. (TIF) [file pcbi.1007530.s005.tif]

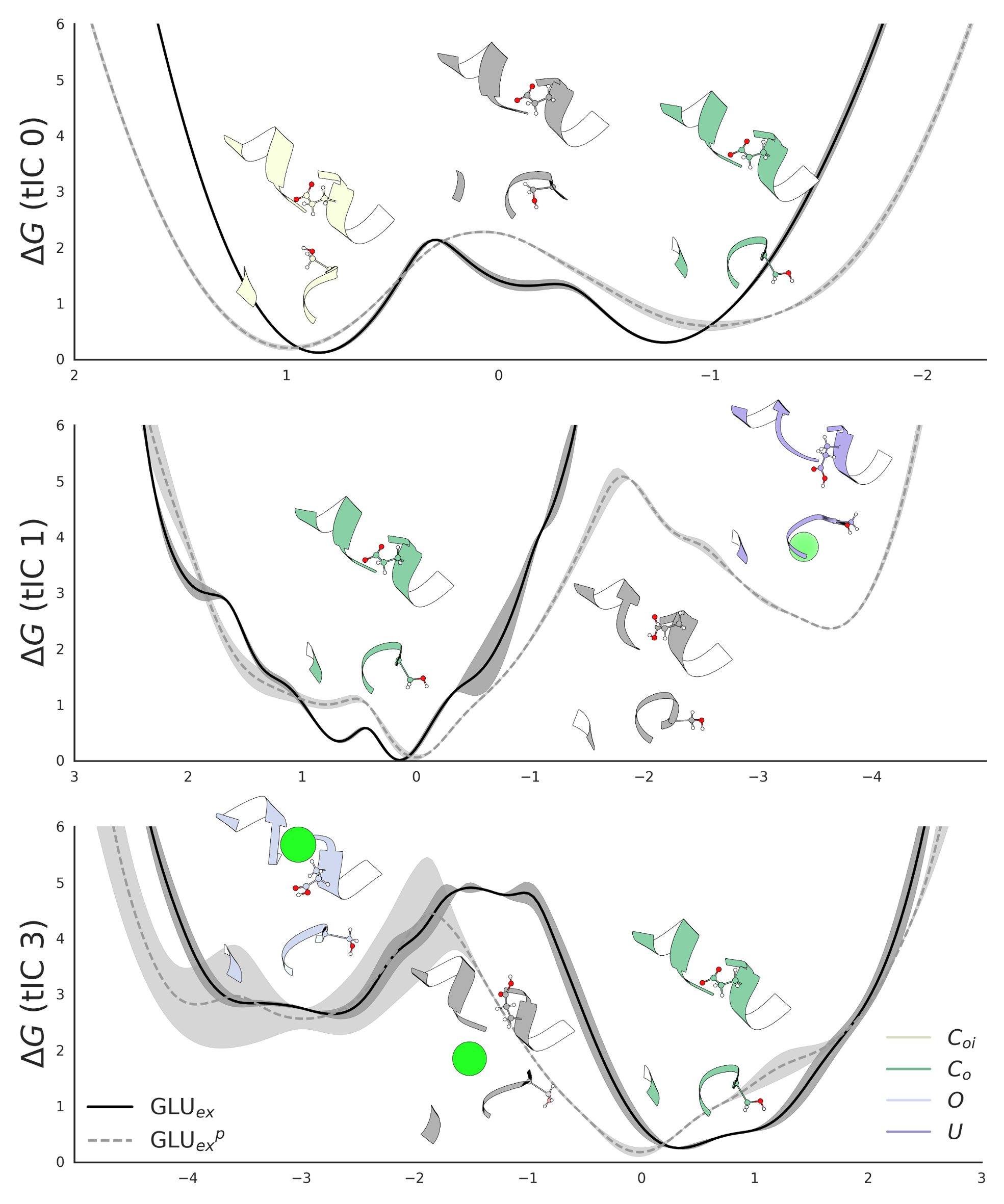

Supplement: S6 Fig — For each coordinate, the transition state conformation is shown in gray. The conformational changes involve rotation of the S168, G169, and I170 residue backbone (tIC 0), as well as the rotameric flip of the GLUex residue (tIC 1, tIC 3). (TIF) [file pcbi.1007530.s006.tif]

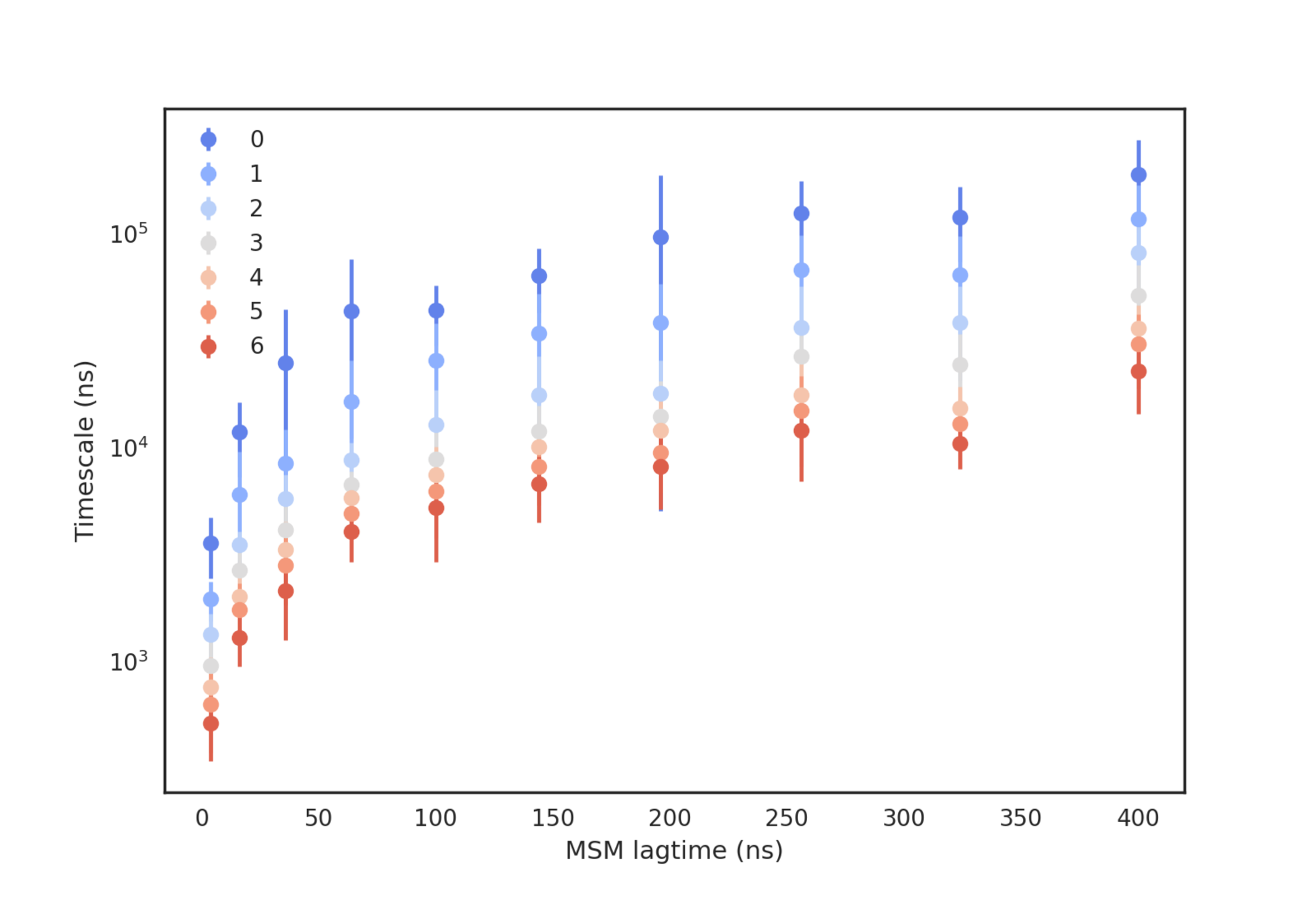

Supplement: S7 Fig — (TIF) [file pcbi.1007530.s007.tif]
