# Supplementary material for: Dynamical model of the CLC-2 ion channel reveals conformational changes associated with selectivity-filter gating
Source: PLoS Comput Biol. 2020 Mar 30;16(3):e1007530. doi: 10.1371/journal.pcbi.1007530 (PMC7145265; doi:10.1371/journal.pcbi.1007530)
Supplement: S1 Table — Each tIC represents a singular vector of the feature set time-lagged autocorrelation matrix. A tIC is therefore given by a linear combination of input features, and can be interpreted by identifying the features with the highest loadings (linear coefficient). The major tICs highlighted in this study are summarized below, where the loadings are listed if they were found to be greater than 2 standard deviations from the mean. (PDF) [file pcbi.1007530.s008.pdf]

featuregroup	featurizer	otherinfo	resnames	resseqs	tIC 0 loading
phi	Dihedral	cos	[GLY, SER]	[167, 168]	0.197249
psi	Dihedral	cos	[GLY, SER]	[168, 169]	0.327256
psi	Dihedral	cos	[GLY, ILE]	[169, 170]	0.944898

featuregroup	featurizer	otherinfo	resnames	resseqs	tIC 1 loading
psi	Dihedral	cos	[GLY, ILE]	[169, 170]	-0.529498
phi	Dihedral	sin	[PHE, ALA]	[464, 465]	-0.190180
psi	Dihedral	sin	[PHE, ALA]	[464, 465]	-0.122220
phi	Dihedral	sin	[GLY, SER]	[168, 169]	-0.117311
psi	Dihedral	cos	[GLY, SER]	[168, 169]	0.093205
phi	Dihedral	sin	[GLY, SER]	[167, 168]	0.105494
chi1	Dihedral	cos	[GLU]	[211]	0.179382

featuregroup	featurizer	otherinfo	resnames	resseqs	tIC 3 loading
phi	Dihedral	cos	[GLY, SER]	[167, 168]	-0.952175
psi	Dihedral	cos	[GLY, SER]	[168, 169]	-0.939586
phi	Dihedral	sin	[GLY, SER]	[167, 168]	-0.530396
phi	Dihedral	sin	[GLY, SER]	[168, 169]	0.268003
chi1	Dihedral	cos	[GLU]	[211]	0.365780
