# Supplementary material for: Dynamical model of the CLC-2 ion channel reveals conformational changes associated with selectivity-filter gating
Source: PLoS Comput Biol. 2020 Mar 30;16(3):e1007530. doi: 10.1371/journal.pcbi.1007530 (PMC7145265; doi:10.1371/journal.pcbi.1007530)
Supplement: S2 Table — Stability of the homology model was determined through calculation of the full protein Ramachandran plot of a MD-relaxed conformation of the homology model. This calculation was done using the UCLA DOE-MBI web server. The Ramachandran plot statistics for the model are very close to that of the template crystal structure (3ORG). (PDF) [file pcbi.1007530.s009.pdf]

Ramachandran plot statistic	3ORG (%)	model (%)
Residues in most favoured regions [A,B,L]	90.1	87.5
Residues in additional allowed regions [a,b,l,p]	9.2	11.1
Residues in generously allowed regions [∼a,∼b,∼l,∼p]	0.4	1.2
Residues in disallowed regions	0.3	0.2
